# Supplementary material for: Deciphering the Physicochemical and Microscopical Changes in Ganoderma boninense-Infected Oil Palm Woodblocks under the Influence of Phenolic Compounds
Source: Plants (Basel). 2021 Aug 28;10(9):1797. doi: 10.3390/plants10091797 (PMC8470138; doi:10.3390/plants10091797)
Supplement: Supplementary file 1 [file plants-10-01797-s001.zip › plants-1305233-supplementary.pdf]

Journal name: Plants: Special Issue: Advances in alternative measures in plant protection

Deciphering the Physicochemical and Microscopical Changes in *Ganoderma boninense*-infected Oil Palm Woodblocks under the Influence of Phenolic Compounds

Arthy Surendran [1,*], Yasmeen Siddiqui [2,*], Khairulmazmi Ahmad [2,*] and Rozi Fernanda [2]

[1] School of Life Sciences, University of Warwick Wellesbourne, Warwick CV35 9EF, UK
[2] Sustainable Agronomy and Crop Protection, Institute of Plantation Studies, Universiti Putra Malaysia, Serdang 43400, Malaysia; rozifernanda86@gmail.com
* Correspondence: Arthy.Surendran@warwick.ac.uk (A.S.); yasmeen@upm.edu.my (Y.S.); khairulmazmi@upm.edu.my (K.A.); Tel.: +60-3-9769-4135 (K.A.)

Table S1: Lower Order Index (LOI) of oil palm wood (with and without treatment) degraded by *G. boninense* at various time intervals

	Biodegradation (days)			
**Phenolic compounds (mM)**	**10**	**30**	**45**	**120**
**Control**	0.97	0.97	0.93	0.86
**BA 1**	0.97	0.97	0.98	0.97
**SA 1**	0.98	0.98	0.93	0.89
**SA 5**	0.98	0.97	0.95	0.90
**SY 1**	0.97	0.99	0.93	0.87
**SY 5**	0.98	0.98	0.99	0.94
**VA 1**	0.99	0.95	0.90	0.83
**VA 5**	0.98	0.96	0.90	0.86
**VA 10**	0.98	0.98	0.95	0.89

BA - benzoic acid, SA - salicylic acid, SY- syringic acid, VA -vanillic acid

Table S2: Total Crystallinity Index (TCI) of oil palm wood (with and without treatment) degraded by *G. boninense* at various time intervals

	Biodegradation (days)			
**Phenolic compounds (mM)**	**10**	**30**	**45**	**120**
**Control**	1.1	1.2	1	0.98
**BA 1**	1.01	0.97	0.95	0.93
**SA 1**	1.04	1.02	1.04	0.99
**SA 5**	1.01	1.01	1	0.98
**SY 1**	1.02	1.02	1.01	1
**SY 5**	1.02	1.02	1.02	1
**VA 1**	1.01	1	1.04	0.96
**VA 5**	1.01	1	1.01	0.98
**VA 10**	1.00	1	1	0.98

BA - benzoic acid, SA - salicylic acid, SY- syringic acid, VA -vanillic acid

Table S3: S/G ratio of oil palm wood (with and without treatment) degraded by *G. boninense* at various time intervals

	Biodegradation (days)			
**Phenolic compounds (mM)**	**10**	**30**	**45**	**120**
**Control**	0.99	0.99	0.98	0.94
**BA 1**	0.99	0.99	0.96	0.92
**SA 1**	1	1	0.99	0.94
**SA 5**	1	1	1.08	0.94
**SY 1**	1	1.1	1	0.94
**SY 5**	1	1.1	1	0.94
**VA 1**	1	1.3	1.2	0.9
**VA 5**	1	1.2	1.1	0.91
**VA 10**	1	1	1	0.93

BA - benzoic acid, SA - salicylic acid, SY- syringic acid, VA -vanillic acid.

Table S4: Percentage of chemical composition of healthy oil palm wood

Chemical Composition	Percentage (%)
Lignin	13.3 ± 0.8
Cellulose	29.9 ± 0.5
Hemicellulose	29.9 ± 0.7
